# Inter-Limb Jump Asymmetries and Their Association with Sport-Specific Performance in Young Male and Female Swimmers

**DOI:** 10.3390/ijerph18147324

**Published:** 2021-07-08

**Authors:** Max I. Phukan, Rohit K. Thapa, Gopal Kumar, Chris Bishop, Helmi Chaabene, Rodrigo Ramirez-Campillo

**Affiliations:** 1 Department of Physical Education Pedagogy, Lakshmibai National Institute of Physical Education, Gwalior 474002, India; induphukan76@gmail.com; 2 Department of Sports Biomechanics, Lakshmibai National Institute of Physical Education, Gwalior 474002, India; rohitthapa04@gmail.com; 3 Department of Exercise Physiology, Lakshmibai National Institute of Physical Education, Gwalior 474002, India; gopalk.kumar8@gmail.com; 4 London Sport Institute, Middlesex University, The Burroughs, London NW44BT, UK; c.bishop@mdx.ac.uk; 5 Faculty of Human Sciences, University of Potsdam, 14467 Potsdam, Germany; chaabanehelmi@hotmail.fr; 6 High Institute of Sports and Physical Education, University of Jendouba, Kef 7100, Tunisia; 7 Department of Physical Activity Sciences, Universidad de Los Lagos, Santiago 8320000, Chile; 8 Centro de Investigación en Fisiología del Ejercicio, Facultad de Ciencias, Universidad Mayor, Santiago 7500000, Chile

**Keywords:** athletic performance, physical fitness, water sport, leg, lower extremity, swimming, youth sports

## Abstract

This study aimed to examine inter-limb jump asymmetries and their association with sport-specific performance in young swimmers. Thirty-eight (male, n = 19; female, n = 19) regional/national level young swimmers (age: 12.3 ± 1.2 years; height: 159.6 ± 8.2 cm; body mass: 52.5 ± 9.2 kg) participated in this study. Inter-limb asymmetries were assessed for single-leg countermovement jump (_SL_CMJ) and single-leg standing long jump (_SL_SLJ). Sport-specific performance was evaluated using front crawl (i.e., 50 m and 25 m) and front crawl kick (i.e., 50 m and 25 m). The kappa coefficient revealed a “slight” level of agreement (Κ = 0.156, 0.184, and 0.197 for female, male, and all, respectively) between the direction of asymmetry for _SL_CMJ and _SL_SLJ, indicating that asymmetries rarely favored the same limb during both tests. A paired sample *t*-test showed a significant difference (*p* = 0.025) between asymmetry scores obtained in _SL_CMJ and _SL_SLJ. No significant difference was found in asymmetry scores between males and females (*p* = 0.099 to 0.977). Additionally, no association between asymmetry scores and sport-specific performance was observed (*p* > 0.05). Our findings highlight the independent nature of inter-limb asymmetries derived from _SL_CMJ and _SL_SLJ among young male and female swimmers. Further, our results suggest no association between jumping asymmetries and sport-specific performance.

## 1. Introduction

Inter-limb asymmetry refers to the difference in function or performance of one limb relative to the other [1]. These limb differences may be due to the asymmetric motor demands, resulting in functional asymmetric adaptions (e.g., greater Achilles tendon stiffness on the take-off leg for long-jumpers; greater muscle mass in the dominant arm of tennis players) [2] to allow the athletes to perform within their sport [3]. Indeed, sport-specific inter-limb asymmetry is common [4]. However, larger inter-limb asymmetry may increase the risk of non-traumatic injuries among athletes [5,6]. In addition, recent studies reported larger inter-limb asymmetry to be associated with reduced physical performance measures in athletes (e.g., linear sprint, change of direction ability, and repeated sprint performance) [7,8,9], compromising technical efficiency in sports activities. Therefore, evaluating inter-limb asymmetries among athletes is crucial [10].

Previous research has reported a significant association between inter-limb asymmetry and performance in soccer and handball players [7,8]. For instance, Bishop et al. [7] found unfavorable significant associations (up to strong) between _SL_CMJ height asymmetry and 10 m sprint (*r* = 0.54–0.87), 20 m sprint (*r* = 0.56–0.79), and 505 change of direction (CoD) speed (*r* = 0.61–0.85) performance in elite academy soccer players aged 15 to 20 years. Similarly, Madruga-Parera et al. [8] found unfavorable associations (moderate) between _SL_CMJ height asymmetry and 8 × 10 repeated sprint ability (*r* = 0.35–0.40). The same authors revealed an unfavorable association (moderate) between single-leg lateral jump asymmetry, V-cut (*r* = 0.31), and CoD speed test (i.e., 2 × 10 m, with 180° turn) (*r* = 0.31) in adolescent handball players aged 16 years. Although previous studies have shown moderate–strong associations between asymmetry and sport-specific performance, others have reported contradictory results [11,12]. Dos’Santos et al. [12] found no significant associations between jumping asymmetries (i.e., _SL_SLJ and single-leg triple hop asymmetry) and CoD speed in multisport collegiate athletes. Similarly, Loturco et al. [11] found no significant associations between vertical jump asymmetries (i.e., _SL_CMJ and single-leg squat jump) and 30 m linear sprint-speed, CoD speed (i.e., zig-zag test), and jump squat in elite female soccer players aged 23 years.

Similarly, a previous study [13] assessed water-based tethered swimming asymmetries (i.e., force production by left and right limb during front crawl) and reported a detrimental association with overall swimming performance. However, water-based assessments are expensive, complex, and time consuming. A more plausible approach would be dry land testing (e.g., CMJ, SLJ). Previous studies [14,15,16] reported association of dry land testing (e.g., mean propulsive power in jump-squat, leg extension, horizontal jump) with front crawl swimming performance (i.e., 25 m and 50 m). Loturco et al. [14] reported a large association between mean propulsive power in jump-squat and 50 m front crawl swimming performance (r = −0.70). Garrido et al. [15] reported association of leg extension exercise with 25 m (ρ = −0.69) and 50 m (ρ = −0.62) front crawl performance. Marinho et al. [16] reported association between horizontal jump and 50 m (r = −0.44) front crawl performance.

In this regard, the relationship between inter-limb asymmetry and performance measures is not yet conclusive [3], and thus warrants further investigation. Furthermore, studies have focused on unilateral jumping inter-limb asymmetries across different sports (e.g., soccer, skateboarders, cricket, basketball, badminton, tennis) and within a specific gender (i.e., either male or female) [17,18,19,20,21,22]. However, inter-limb jump asymmetry (i.e., using dry land tests such as CMJ and SLJ) among young male and female swimmers and their relationship with sport-specific performance has received little attention in the literature.

Therefore, the primary aim of this study was to examine inter-limb jump asymmetry (i.e., both vertical and horizontal) in young male and female swimmers. The second purpose was to identify the effect of inter-limb asymmetry on sport-specific performance (e.g., front crawl) in young swimmers. We hypothesized significant vertical and horizontal inter-limb jump asymmetry differences between young male and female swimmers, with an unfavorable association with sport-specific performance [13].

## 2. Materials and Methods

### 2.1. Participants

Forty-four young swimmers who regularly compete in regional/national level events initially agreed to participate in the study. Few participants were above 15 years of age (i.e., youth athletes [23]). Therefore, to obtain a more homogenous sample of young athletes, participants above 15 years old (n = 6) were not included. Therefore, thirty-eight young swimmers (19 males and 19 females; age: 12.3 ± 1.2 years; height: 159.6 ± 8.2 cm; body mass: 52.5 ± 9.2 kg; swimming experience: 5.4 ± 0.8 years) were retained. A posteriori power calculation using G*Power software (v.3.1.9.4, University of Kiel, Kiel, Germany) with ɑ = 0.05, n = 38 and effect size = 0.303 (correlation coefficient _SL_SLJ and 50 m front crawl performance) estimated power of 0.61. All included athletes had participated in regional and/or national level competitions in the previous year; and were free of any injury in the previous six months that could limit the unilateral jump performance or swimming performance. The participants practiced multiple swimming styles. In addition, participants were engaged in multiple recreational sports activities (not more than 2 h/week) as a part of their training curriculum. The experimental procedure of the study was explained to participants and their parents. Thereafter, written informed consent was provided by their parents or legal guardians and participants gave their assent. The study was approved by the Lakshmibai National Institute of Physical Education (Academic/380/1317) and conducted per the protocol laid down under the declaration of Helsinki.

### 2.2. Procedures

The data collection was conducted over a month in three different swimming training centers. All participants performed ≥7 familiarization sessions with the _SL_CMJ and _SL_SLJ tests before the collection of the data. All testing sessions were conducted outdoors. The mean temperature, humidity, and wind velocity were 22.7 ± 1.5 °C, 69.0 ± 8.5%, and 6.0 ± 1.0 km/h, respectively. All tests were conducted between 15:00 h and 18:00 h and participants were instructed to avoid strenuous activity 24 h before data collection, and heavy meals or caffeinated drinks 3 h before data collection.

### 2.3. Unilateral Jump Tests

The two unilateral jump tests were conducted to quantify the asymmetry on both horizontal (i.e., _SL_SLJ) and vertical (i.e., _SL_CMJ) components. The tests were conducted (in the aforementioned order) after a dry land warm-up of ~10 min. The warm-up included slow jogging at 60% of self-estimated intensity, arm swings, arm rotations, forward lunges, lateral lunges, inchworm, countermovement jumps, and standing broad jumps.

The _SL_SLJ test was conducted on the floor (concrete pool arena) with a marked tape attached. Participants were instructed to keep their non-jumping leg at the middle of the shin (i.e., medial malleolus) and jump as far as possible along the direction of the tape following a self-selected countermovement. The jumping leg was positioned with the toes just behind the 0 m jumping line (i.e., starting line). Trials involving swinging of the non-jumping leg before the jump were disallowed, and a new trial was conducted after a recovery period of ~30 s. The participants were instructed to jump as far as possible forward and to land on the same leg. The horizontal distance between the starting line and the heel of the landing foot was recorded to the nearest centimeter. Three trials were conducted for each leg, with ~30 s of recovery between trials with the same leg. The average of the three trials for each leg was selected for the analysis, and asymmetry within the _SL_SLJ test was determined as the average of left and right leg performance [1].

The _SL_CMJ test was conducted after the _SL_SLJ test, with ≥1 min recovery period. The _SL_CMJ was conducted with arms akimbo. Participants were instructed to jump for maximal height, after a self-selected knee flexion angle (i.e., countermovement). The participants were instructed to keep their non-jumping leg at the middle of the shin (i.e., medial malleolus) of the jumping leg, without swinging the non-jumping leg. Jump height generated during the _SL_CMJ was measured with a validated iOS application, My Jump 2, installed on an Apple iPad 8th generation (Apple Inc., California, USA) with a 120 Hz high-speed camera at a quality of 720 p. The camera was directed as low as possible facing each athlete in the frontal plane ~2 m away to best record jump performance. Three trials were conducted for each leg, with ≥30 s of rest between trials with the same leg. The average of the three trials for each leg was selected for the analysis, and asymmetry within the _SL_CMJ test was determined as an average of left and right leg performance [1].

### 2.4. Asymmetry Index

The asymmetry index was calculated using the equation ((dominant limb–non-dominant limb)/dominant limb) × 100 [1], where the dominant limb was the one with the highest jump height or longest jump distance.

### 2.5. Sport-Specific Swimming Tests

On a separate day from that of the _SL_SLJ and _SL_CMJ tests, sport-specific swimming performance was measured through the front crawl (25 m and 50 m), and front crawl kick with push (25 m and 50 m). A swimming-specific warm-up was performed before testing. The warm-up included 200 m free swim, 200 m kick-drill, 200 m free-back, 2 × 50 m build-up, and 2 × 50 m build-down. Front crawl tests were performed with a diving start, whereas front crawl kick with push trials involved a push-off from the wall. These testing procedures during the front crawl kick with push were intended to better assess the effect of lower limbs by minimizing the influence of hand swing during diving. The tests were conducted in a standard 50 m swimming pool with the 50 m tests conducted on the length of the pool, and the 25 m tests conducted on the breadth of the pool. The participants were not allowed to drift forward or backward before initiating the start during the diving start test (i.e., front crawl 25 m and 50 m). For the dive start, the signal for the timekeeper was the movement as the swimmer’s feet left the block, whereas for the water start with push-off, the swimmer’s first lower limb movement was used as an indicator to start timing. The final signal for the timekeepers to stop recording timing was the moment when the first hand of the swimmer touched the wall. Two independent timekeepers were assigned to record the timing of each trial with a stopwatch. The between timekeepers intraclass correlation coefficients (ICCs) for all tests were ≥0.99. After completion of each trial, swimmers were instructed to swim back slowly to the starting point at ~50% of their maximal 100 m speed. Thereafter, a ~3 min passive recovery period was allowed. The average of two trials was used for further statistical analysis. All the tests were conducted under the supervision of qualified swimming coaches.

### 2.6. Statistical Analyses

All statistical analyses were conducted using IBM SPSS version 20 (IBM, New York, NY, USA). Data are presented as mean ± standard deviation (SD). The assumptions of normality of the data were verified using the Shapiro–Wilk test for the parametric test, and data violating the normality assumptions (i.e., _SL_SLJ) were analyzed using a non-parametric test (i.e., Mann–Whitney U-test and Spearman’s rho correlations). Asymmetry direction was calculated for each participant using the “IF” function, i.e., using the formula “ = asymmetry score*IF(left < right,1,−1)” in Microsoft Excel, and the level of agreement (i.e., consistency) between tests (i.e., _SL_CMJ and _SL_SLJ) for asymmetry direction was calculated using kappa statistics and interpreted as poor (<0), slight (0.01–0.20), fair (0.21–0.40), moderate (0.41–0.60), substantial (0.61–0.80), and almost perfect (0.81–0.99) [24]. Test-retest reliability for performance variables (i.e., _SL_SLJ, _SL_CMJ, sport-specific swimming tests) was determined using the coefficient of variation (CV = (standard error of measurement/subjects mean score) × 100), and ICC (two-way random effects model). The CV values <10% were considered acceptable [25]. The ICC between trials was interpreted as poor (<0.5), moderate (0.5–0.75), good (0.75–0.9), and excellent (>0.9) reliability based on the lower bound of the 95% confidence interval (CI; ICC_95%CI lower bound_) [26].

A paired *t*-test was used to analyze the difference between the magnitudes of asymmetry between _SL_SLJ vs. _SL_CMJ. An independent *t*-test and Mann–Whitney U-test were also conducted to determine the difference between asymmetry scores between males and females. Pearson’s r and Spearman’s rho correlations were conducted to determine the relationship between asymmetry scores and swimming-specific tests. The magnitude of the correlation between test measures was interpreted as trivial (≤0.1), small (0.1–0.3), moderate (0.3–0.5), large (0.5–0.7), very large (0.7–0.9), and almost perfect (0.9–1.0) [27]. The level of significance was set at *p* ≤ 0.05.

## 3. Results

Mean test scores, inter-limb asymmetries, and reliability outcomes are presented in Table 1. All jump tests reported good-to-excellent reliability (ICC_95%CI lower bound_ = 0.87 to 0.93) and acceptable variability (CV = 5.2 to 9.6%) (Table 1). Similarly, good-to-excellent reliability (ICC_95%CI lower bound_ = 0.76 to 0.92) and almost acceptable variability (CV = 5.0 to 11.2%) were shown when the swimmers were grouped based on gender (Table 1). All sport-specific tests reported good reliability (50 m front crawl: ICC_95%CI lower bound_ = 0.82; 25 m front crawl: ICC_95%CI lower bound_ = 0.86; 50 m kick: ICC_95%CI lower bound_ = 0.78; 25 m kick: ICC_95%CI lower bound_ = 0.79).

**Figure 1:**
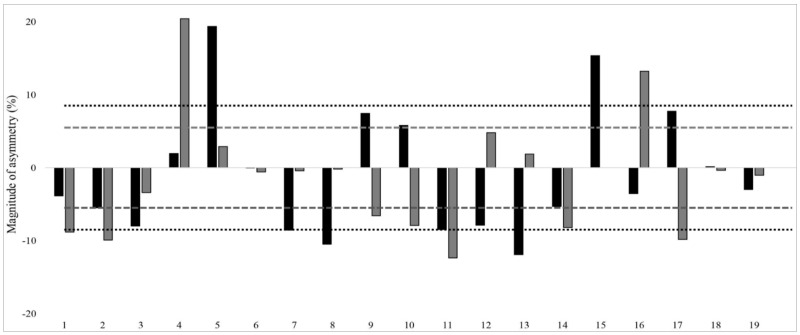
Inter-individual single-leg countermovement jump (_SL_CMJ) and single-leg standing long jump (_SL_SLJ) asymmetry scores among young male swimmers (n = 19). Note: Black and grey bars denote _SL_CMJ and _SL_SLJ asymmetry scores, respectively. Score above and below 0 indicates asymmetry is favored on the right and left leg, respectively. Round and squared dotted lines indicate the average coefficient of variation for _SL_CMJ (8.5%) and _SL_SLJ (5.5%), respectively.

**Figure 2:**
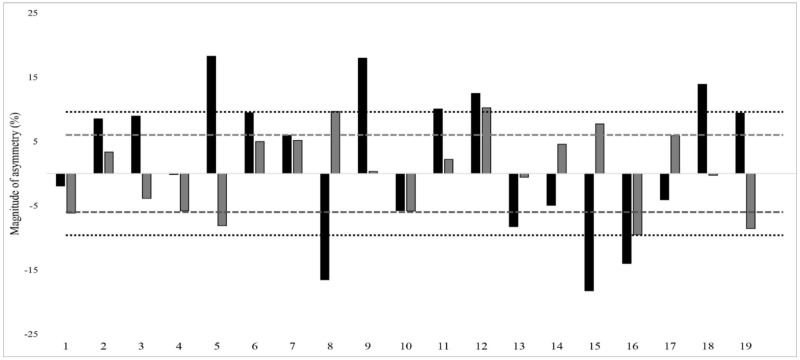
Inter-individual single-leg countermovement jump (_SL_CMJ) and single-leg standing long jump (_SL_SLJ) asymmetry scores among young female swimmers (n = 19). Note: Black and grey bars denote _SL_CMJ and _SL_SLJ asymmetry scores, respectively. Score above and below 0 indicates asymmetry is favored on the right and left leg, respectively. Round and squared dotted lines indicate the average coefficient of variation for _SL_CMJ (9.5%) and _SL_SLJ (6%), respectively.

A kappa coefficient value of 0.197 (overall), 0.184 (male) and 0.156 (female) was obtained between _SL_CMJ and _SL_SLJ asymmetry direction, which shows that asymmetries rarely favored the same side between the two jump tests, indicating that limb dominance is jump test-dependent. Individual jump asymmetry data with the direction of asymmetry for both tests are presented in Figure 1 and Figure 2.

A paired *t*-test reported a significant difference between _SL_CMJ and _SL_SLJ asymmetry magnitude (*p* = 0.025) with higher scores for _SL_CMJ compared with _SL_SLJ. In addition, an independent *t*-test and Mann–Whitney U test reported no significant difference between males and females in _SL_CMJ (*p* = 0.099) and _SL_SLJ (*p* = 0.977), respectively.

The correlation between jumping asymmetries and sport-specific swimming performance is presented in Table 2. No significant correlations were found between asymmetry scores and sport-specific swimming performance.

## 4. Discussion

This study aimed to (i) examine inter-limb jump asymmetries using _SL_CMJ and _SL_SLJ, and (ii) investigate the effect of inter-limb asymmetries on sport-specific swimming performance (e.g., front crawl) in young male and female swimmers. The main findings showed no significant difference in the magnitude of asymmetry between males and females. In addition, results showed no association between jumping-based asymmetries and sport-specific swimming performance in young swimmers. Further, the asymmetries rarely favored the same side between vertical (_SL_CMJ) and horizontal (_SL_SLJ) jumping tests.

When asymmetry scores of all participants were considered, there was a significantly larger asymmetry in the _SL_CMJ than _SL_SLJ. This finding is in line with previous studies [8,9,28]. Lockie et al. [28] reported larger inter-limb differences for the _SL_CMJ (10.4%) than for _SL_SLJ (3.3%) in male team sport athletes aged 23 years. Additionally, Bishop et al. [9] conducted a study of elite youth female soccer players and reported significantly larger asymmetries for _SL_CMJ (12.5%) than for _SL_SLJ (6.8%). Similar observations were shown by Madruga-Parera et al. [8], who reported larger asymmetry for _SL_CMJ (11.2%) than _SL_SLJ (6.4%) in adolescent male handball players aged 16 years. Furthermore, a previous study in elite youth female soccer players aged 10 years by McCubbine et al. [29] suggested that _SL_CMJ (ICC_95%CI lower bound_ = 98 both right and left leg) affords higher accuracy at detecting asymmetries compared with _SL_SLJ (ICC_95%CI lower bound_ = 0.40–0.48) due to its ability to expose greater inter-limb asymmetries and stronger reliability than the horizontal tests. Overall, although our findings indicate that both _SL_CMJ and _SL_SLJ tests may be a useful (and reliable) option for practitioners for quantifying limb differences for young swimmers aged 10 to 15 years, _SL_CMJs and _SL_SLJs should not be used interchangeably.

Bishop [30] also suggested that the “direction of asymmetry” in conjunction with its magnitude could help in understanding which limb performed better as an absolute positive value of asymmetry would fail to identify the same. Therefore, the “asymmetry direction” method is used to identify consistency (i.e., by using the kappa coefficient method [31]) of jumping asymmetries favoring the same side (i.e., left vs. right) between different tests [8,32,33]. Our results show only a “slight” level of agreement (kappa = 0.197) for the consistency of asymmetry direction between _SL_CMJ and _SL_SLJ among young swimmers, which is in agreement with previous studies [8,32,33]. Madruga-Parera et al. [8] detected “poor” to “slight” levels of agreement between _SL_CMJ, _SL_SLJ, and single-leg lateral jump in male adolescents handball players aged 16 years. Similarly, Loturco et al. [33] detected no inter-relationships between asymmetries calculated from isokinetic dynamometry, tensiomyography, CMJ, and squat jumps. Further, Bishop et al. [32] detected a “slight” level of agreement when reporting side consistency of peak force asymmetries between _SL_CMJ and _SL_SLJ. Therefore, the findings of our study further support the task-specific nature of asymmetries and thus practitioners should not solely depend on one screening method for assessing asymmetry. Indeed, a more pragmatic approach would be to interpret both vertical and horizontal jump test results as separate entities. In addition, a previous study by West et al. [34] also reported different peak vertical (1541 ± 306 N) and horizontal force (809 ± 223 N) during a freestyle sprint start, and showed its association with 15 m swim-start performance, thereby confirming the necessity of both vertical and horizontal assessments for swimmers.

Our findings also showed no significant association of inter-limb jump asymmetries with swimming sport-specific performance. Although this is the first study to examine the association of dry land jump asymmetry and swimming performance in young athletes of both sexes, two previous studies assessed asymmetry using a tethered swimming test (i.e., using strain gauge) and reported similar [35] and contradicting [13] findings to ours. Morouço et al. [35] analyzed asymmetries during front crawl tethered swimming and reported no association of inter-limb asymmetries with short-sprint swimming performance. In contrast, Dos Santos et al. [13] examined asymmetries during front crawl tethered swimming and reported that an increase in inter-limb differences in force production may be detrimental to overall swimming performance. Of note, unlike our study, the aforementioned studies [13,35] assessed asymmetry during a static swimming test and quantified asymmetry via the force produced by each segment (i.e., right or left) during the swimming test. Considering the practical aspects associated with dry land _SL_CMJ and _SL_SLJ asymmetry tests, future studies may consider examining its association with swimming-specific asymmetry test (i.e., using a tethered swimming test).

Similar to our findings, no association of jumping asymmetries and performance measures (e.g., linear sprints, CoD) were found in soccer, handball, and multi-sport collegiate athletes [8,11,12]. Dos’Santos et al. [12] reported no association of single-leg hop and single-leg triple hop with the modified 505 agility test (r = 0.22 to −0.29), and 90° cutting task (r = −0.08 to 0.35) among male collegiate team sport athletes (i.e., soccer, rugby, and cricket) aged 22 years. Loturco et al. [11] also found no association of inter-limb jump asymmetries (i.e., _SL_CMJ and single-leg squat jump) and performance measures (5, 10, 20, and 30 m linear sprint, zigzag CoD, and muscle power test using the jump squat) in elite female soccer players aged 23 years. Madruga-Parera et al. [8] also reported no association of _SL_CMJ and _SL_SLJ with 20 m, CoD with 180° turns, and CoD with 45° turns in male adolescent handball players aged 16 years. The lack of association between jump asymmetries and performance in our study may be related to the fact that swimmers perform symmetric movement of the limbs. Continued participation in swimming might have developed the ability to maintain alignment by refining actions such as propulsion, breathing, recovery, and entry [36], thereby minimizing the effects of limb asymmetry during jumps in swimming-specific performance.

Further, meaningful asymmetries were found in our study. It is possible that these findings may relate to the age of the swimmers (10–15 years), who are still in their growth and developmental stages. Similar meaningful asymmetries were observed in male youth handball players aged 16 years (i.e., _SL_CMJ and _SL_SLJ asymmetry = 11.2 ± 8.4% and 8.3 ± 7.5%, respectively) and basketball athletes aged 18 years (_SL_CMJ asymmetry = 10.6 ± 8.6%) [8,22]. The asymmetry scores for individual athletes in _SL_CMJ and _SL_SLJ tests are presented in Figure 1 and Figure 2, with dotted lines indicating the CV for each test. The relevance of this CV for each test is that when asymmetry scores surpass the CV, the imbalances are greater than the test variability and can be considered to be real [1,37]. The individual asymmetry scores were greater than the CV among 15 swimmers and 19 swimmers in _SL_CMJ and _SL_SLJ, respectively. Therefore, ≤50% of the swimmers had real inter-limb differences during unilateral jump testing. Further studies may consider investigating how such results are modified as athletes mature and their interaction with training.

Some limitations are acknowledged in our study. Firstly, only young athletes ≤15 years were included in the study. Therefore, the study results are not conclusive for >15 year old athletes (i.e., more mature youths or those who are no longer in the growth phase). Secondly, the swimming-specific tests were conducted only on short distances (i.e., 25 m and 50 m). Therefore, future studies including longer distances are encouraged. Thirdly, the power obtained in the study was low (i.e., 0.61); therefore, future research should include larger sample size. Finally, the inter-limb asymmetry was evaluated in a single session, not in multiple sessions as previously recommended [38]. Future research should evaluate inter-limb asymmetry in multiple sessions to ensure direction of asymmetry favors the same limb.

## 5. Conclusions

The findings highlight the independent nature of inter-limb asymmetries derived from _SL_CMJ and _SL_SLJ among young male and female swimmers. Further, our results suggest no association between jumping asymmetries and sport-specific performance.

## Figures and Tables

**Table 1:** Asymmetry scores in single-leg countermovement jump (_SL_CMJ) and single-leg standing long jump (_SL_SLJ) in young male and female swimmers.

Variable		Mean ± SD (m)	Asymmetry %	Intraclass Correlation Coefficient (95% CI)	Coefficient of Variation (%)
Overall (n = 38)	_SL_CMJ (left)	0.10 ± 0.02	8.5 ± 5.3 *	0.93 (0.87–0.96)	9.6
	_SL_CMJ (right)	0.10 ± 0.03		0.95 (0.91–0.97)	8.0
	_SL_SLJ (left)	1.22 ± 0.21	5.7 ± 4.5	0.97 (0.93–0.98)	5.2
	_SL_SLJ (right)	1.21 ± 0.21		0.94 (0.89–0.97)	6.4
Male (n = 19)	_SL_CMJ (left)	0.11 ± 0.02	7.1 ± 4.9	0.89 (0.76–0.95)	9.0
	_SL_CMJ (right)	0.11 ± 0.02		0.94 (0.86–0.97)	8.0
	_SL_SLJ (left)	1.34 ± 0.21	6.0 ± 5.6	0.97 (0.92–0.99)	5.0
	_SL_SLJ (right)	1.32 ± 0.21		0.93 (0.84–0.97)	6.0
Female (n = 19)	_SL_CMJ (left)	0.09 ± 0.02	9.9 ± 5.5	0.93 (0.85–0.97)	11.0
	_SL_CMJ (right)	0.09 ± 0.02		0.95 (0.89–0.98)	8.0
	_SL_SLJ (left)	1.09 ± 0.12	5.4 ± 3.1	0.89 (0.76–0.96)	6.0
	_SL_SLJ (right)	1.10 ± 0.13		0.89 (0.76–0.95)	6.0

CI: confidence interval; m: meter; SD: standard deviation; * significant difference with _SL_SLJ asymmetry at *p* = 0.025.

**Table 2:** Correlation coefficient between asymmetry scores and swimming performance.

Asymmetry %		50 m Front Crawl	25 m Front Crawl	50 m Kick	25 m Kick
Overall (n = 38)	_SL_CMJ	0.296	0.247	0.087	−0.007
	_SL_SLJ	0.303 ^ρ^	0.285 ^ρ^	−0.011 ^ρ^	0.023 ^ρ^
Male (n = 19)	_SL_CMJ	0.361	0.288	0.016	0.135
	_SL_SLJ	0.321 ^ρ^	0.268 ^ρ^	0.032 ^ρ^	0.230 ^ρ^
Female (n = 19)	_SL_CMJ	0.144	0.168	0.244	−0.149
	_SL_SLJ	0.330	0.265	0.016	0.311

ρ: denotes Spearman’s rho (i.e., non-parametric test) correlations. _SL_CMJ: single-leg countermovement jump; _SL_SLJ: single-leg standing long jump.

## Data Availability

The datasets generated and analyzed for this study can be obtained from the corresponding author.
